# The shaping of gut immunity in cirrhosis

**DOI:** 10.3389/fimmu.2023.1139554

**Published:** 2023-04-14

**Authors:** Leticia Muñoz, Esther Caparrós, Agustín Albillos, Rubén Francés

**Affiliations:** ^1^ Departamento de Medicina y Especialidades Médicas, Universidad de Alcalá, Alcalá de Henares, Madrid, Spain; ^2^ Centro de Investigación Biomédica en Red de Enfermedades Hepáticas y Digestivas (CIBERehd), Instituto de Salud Carlos III, Madrid, Spain; ^3^ Grupo de Inmunobiología Hepática e Intestinal, Departamento Medicina Clínica, Universidad Miguel Hernández, San Juan, Spain; ^4^ Instituto de Investigación Sanitaria ISABIAL, Hospital General Universitario de Alicante, Alicante, Spain; ^5^ Departamento de Gastroenterología y Hepatología, Hospital Universitario Ramón y Cajal, Instituto Ramón y Cajal de Investigación Sanitaria (IRYCIS), Madrid, Spain; ^6^ Instituto de Investigación, Desarrollo e Innovación en Biotecnologiía Sanitaria de Elche (IDiBE), Universidad Miguel Hernández, Elche, Spain

**Keywords:** cirrhosis, gut permeability, inflammation, bacterial translocation, microbiota

## Abstract

Cirrhosis is the common end-stage of chronic liver diseases of different etiology. The altered bile acids metabolism in the cirrhotic liver and the increase in the blood-brain barrier permeability, along with the progressive dysbiosis of intestinal microbiota, contribute to gut immunity changes, from compromised antimicrobial host defense to pro-inflammatory adaptive responses. In turn, these changes elicit a disruption in the epithelial and gut vascular barriers, promoting the increased access of potential pathogenic microbial antigens to portal circulation, further aggravating liver disease. After summarizing the key aspects of gut immunity during homeostasis, this review is intended to update the contribution of liver and brain metabolites in shaping the intestinal immune status and, in turn, to understand how the loss of homeostasis in the gut-associated lymphoid tissue, as present in cirrhosis, cooperates in the advanced chronic liver disease progression. Finally, several therapeutic approaches targeting the intestinal homeostasis in cirrhosis are discussed.

## 1 Background

Cirrhosis is an evolving liver disease that leads to portal hypertension and hepatic insufficiency. During cirrhosis, the composition of intestinal microbiota is dynamically altered. This microbiota state, known as dysbiosis, induces an increased intestinal permeability and gut barrier dysfunction. In these conditions, the homeostatic contribution of the liver and the brain to gut immunity that is established through the gut-liver-brain axis is then affected, accelerating disease decompensation and complications (1, 2). This review outlines intestinal immunity characteristics in the homeostatic condition and how metabolites derived from liver and brain during cirrhosis contribute to disturbing the gut tissue microenvironment, which further contributes to progression of advanced chronic disease.

### 1.1 Intestinal immune milieu in homeostasis

**Figure 1:**
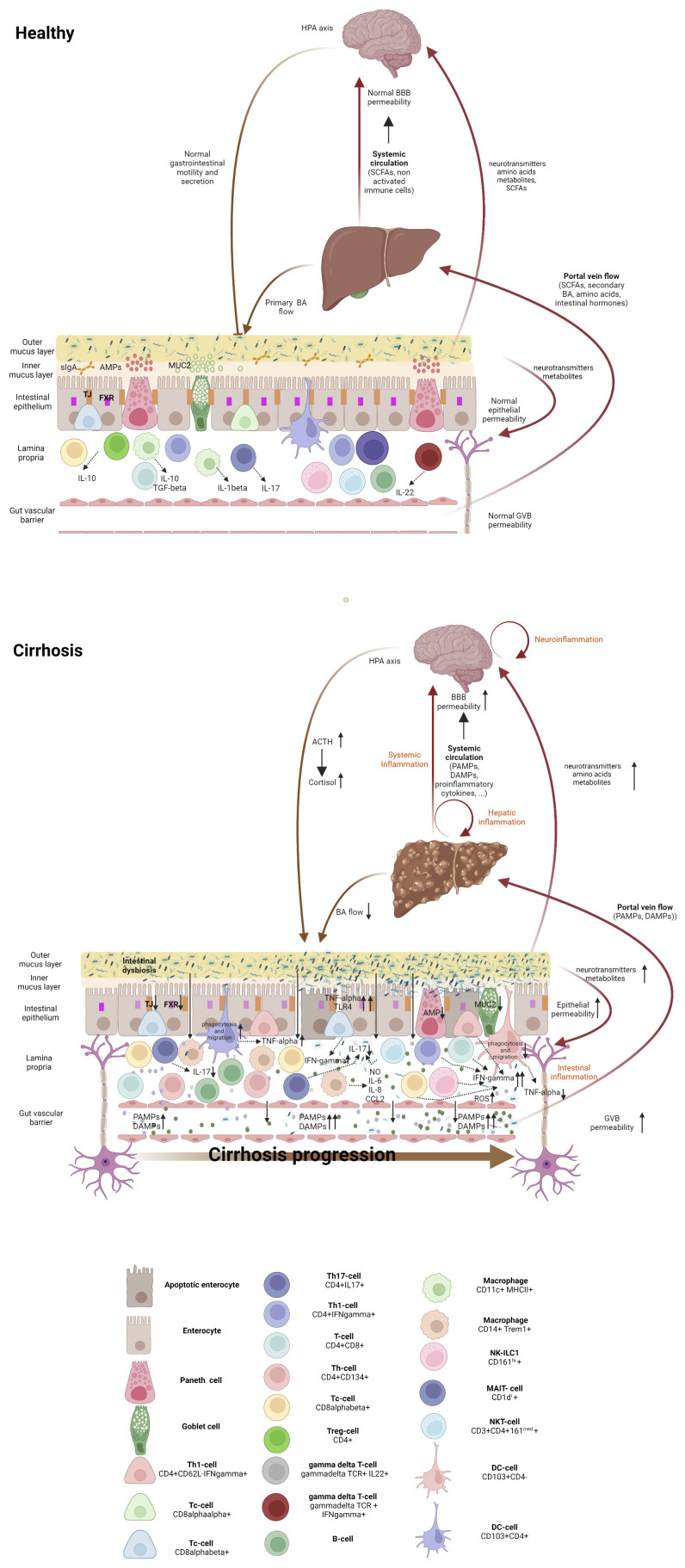
Gut-liver-brain axis in health and cirrhosis, highlighting the intestinal immune milieu. The intestinal epithelial barrier is a multi-layered structure that separates bacteria in the intestinal lumen from the systemic circulation. In healthy individuals, eubiosis, a tolerant immune system and an efficient innate antibacterial defense maintain the integrity of the epithelial and vascular barriers. Cirrhosis is associated with an altered microbiome which results from decreased bile flow, with deficient levels of primary but increased of secondary bile acids, together with intestinal hypomotility. Gut dysbiosis drives bacterial colonization of the inner mucous layer of the intestine, which facilitates the interaction of bacteria with the immune system, resulting in intestinal inflammation with recruitment and activation of immune system cells. Composition and severity of intestinal inflammation change with cirrhosis progression. Intestinal inflammation in the compensated stage is featured by activated innate cells, including DCs producing TNF-α and showing increased phagocytic and migratory abilities, along with the activation of the adaptive immune system, mainly located in the intraepithelial compartment. In the decompensated stage, intestinal immune system derangement is characterized by non-activated DCs, with lowered TNF-α secretion, and deficient phagocytosis and migration abilities, as well as expansion of activated macrophages and Th1 lymphocytes with concomitant Th17 reduction. Inflammation results in the release of proinflammatory cytokines and ROS, which worsens epithelial and the vascular barrier damage, hyperpermeability and access of bacterial products to the liver (via portal vein Flow) and to the systemic circulation. Elevated systemic levels of proinflammatory cytokines, PAMPs and DAMPs accelerate cirrhosis progression and increase the blood-brain-barrier permeability, inducing neuroinflammation (astrocyte swelling, activated microglia and immune system infiltration), which activates hypothalamic-pituitary-adrenal axis, and affects intestinal barrier integrity. Within the gut, the altered microbiota can produce neurotransmitters, amino acids and microbial metabolites. These metabolites can travel through portal circulation to interact with the host immune system, influence metabolism and/or affect local neuronal cells of the enteric nervous system and afferent pathways of the vagus nerve that signal directly to the brain. DC, dendritic cell; Treg, regulatory T cells; Tc, cytotoxic T cell; Th, helper T cell; ILC, innate lymphoid cell; NK cell, natural killer cell; NKT cell, natural killer T cell, MAIT cell, mucosal-associated invariant T cell, AMP, antimicrobial peptides; MUC-2, mucin-2; FXR, Farnesoid X receptor, TJ, Tight junction, BA, bile acid; NO, nitric oxide; IL-, interleukin; CCL2, C-C motif ligand 2; PAMPs, pathogen-associated molecular patterns; 318 DAMPs, damage-associated molecular patterns, ROS, radical oxygen species; TNF-γ, tumor necrosis factor-alpha; IFN-γ, interferon γ; ACTH, adrenocorticotrophic hormone; BBB, blood-brain barrier; HPA, hypothalamic-pituitary-adrenal.

Multiple barriers separate the commensal microbes in the gut from blood, avoiding bacteria entering the systemic circulation. From the lumen inwards, tissue arrangement comprises the mucus layer, the epithelial barrier, the immune system layer, and the gut vascular barrier (GVB) (3) (**Figure 1**).

The mucus layer acts as a frontline barrier separating the intestinal lumen from underlying tissues. Mucins comprising the outer thick mucus layer, which help the adhesion of microorganisms and their products, are produced by goblet and epithelial cells, and facilitate the opsonizing activity of plasma cell-derived secretory Immunoglobulin A (sIgA) (**Table 1**). Mucins can be found either as membrane-attached, such as MUC1, MUC3, MUC4, MUC13, and MUC17, or as secreted gel-forming mucins, like MUC2, MUC5AC, MUC5B, and MUC6 (47). Particularly, MUC2 offers static protection and restricts the potential activation of dendritic cells (DC), conferring them a tolerogenic state through stimulation of interleukin 10 (IL-10) production and TNF-α signaling inhibition (48).

**Table 1:** Role of immune cells involved in gut homeostasis and abnormalities produced in cirrhosis.

Cell type		Immune function	References	Intestinal mucosa abnormalities in cirrhosis	References
**Intestinal epithelial cells**	**Enterocytes**	Form TJ structures; produce antimicrobial molecules (Reg3γ and Lypd8); express pIgR	(4)	Shedding and apoptosis, decreased TJs and AMPs Reg3β and Reg3γ	(5–7)
	**Paneth cells**	Produce AMPs α-defensin 5 (HD5), HD6 and Reg3γ	(4, 8, 9)	Defective production of AMPs (α-defensins and Reg3 proteins)	(6, 7, 10, 11)
	**Goblet cells**	Produce mucin and anti-inflammatory molecules (trefoil factor 3, RELMbeta)	(4, 12)	Loss of goblet cells and decreased MUC2 resulting in reduced mucous layer thickness	(13)
	**Microfold (M) cells**	Uptake of antigens	(4, 14)		*^N/R^*
	**Tuft cells**	Sense luminal helminths			
**sIgA (produced by plasma cells)**		Binding and retention of bacteria in the intestinal lumen	(15)	Reduced synthesis in the jejunum and diminished fecal content	(5, 16, 17)
**Neutrophils**		Elimination of translocated microbes, facilitation of mucosal healing, recruitment of other immune cells	(18, 19)		*^N/R^*
**Macrophages**		Bactericidal activity, transfer of acquired antigen to DC for presentation to T cells, production of immunoregulatory cytokines IL-10 and TGF-β	(20)	Activated CD14+Trem1+iNOS+ secreting IL-6, IL-8, NO and MCP-1	(21)
**Dendritic cells**		Maintenance of tolerogenic state; initiation and differentiation of adaptive immune responses	(22)	Deficient phagocytosis, migration capacity, and TNF-α production	(23)
**Eosinophils**		Cytotoxic effect, modulation of B and T cells	(24, 25)		*^N/R^*
**Mast cells**		Innate response, antigen clearance, release of histamine, proteases, and prostaglandins	(26, 27)		*^N/R^*
**ILC1 (including NK cells)**		Cytotoxicity, macrophage activation	(28, 29)	Expanded showing increased IFN-γ production	(5)
**ILC2**		Immunity to helminths	(28)		*^N/R^*
**ILC3**		Host defense against extracellular bacteria and fungi	(28)	Reduced IL-22 production by ILC3s which promotes diminished intestinal Reg3γ expression, resulting in bacterial translocation to the liver	(30)
**iNKTs**		Response to lipid antigen, production of cytokines and chemokines	(28)	Expanded in intestinal lamina propria, but reduced IL-17 production	(5)
**Tgamma-delta**		Defense against infection and wound healing	(31)	Expanded in intestinal lamina propria	(5)
**MAIT cells**		Modulation of host-microbial interplay, antibacterial immunity	(32, 33)		*^N/R^*
**Th CD4+**	**Th17**	Neutrophilic inflammation, response to extracellular bacteria and fungi	(34–39)	Diminished in the lamina propria	(5)
	**Th1**	Monocytic inflammation, response to intracellular bacteria, protozoans, and viruses		Increased production of IFN-γ in the small intestine	(5)
	**Th22**	Mucosal host defense, secretion of beta-defensins	(40)		*^N/R^*
	**Treg**	Tissue homeostasis and tolerogenic cytokine production	(41–44)	Expanded in the lamina propria	(5)
**Tc CD8+**		Cytotoxic activity	(45)	Expanded showing high IFN-γ production	(5)
**Memory T cells**	**Central memory T cells**	Re-exposure with their cognate antigen recirculating between secondary lymph organs and blood	(46)	Expanded in the small intestine	(5)
	**Effector memory T cells**	Re-exposure with their cognate antigen recirculating between peripheral circulation and tissue		Expanded in the small intestine	

N/R, none reported.

The physical barrier integrated by mucins is reinforced by antimicrobial peptides (AMPs), that are produced by enterocytes and Paneth cells, and by microbial metabolites from commensal microbiota (49). AMPs act as efficient barrier against enteric pathogens (50). Cathelicidins, defensins and the regenerating islet-derived protein III-gamma (Reg3γ) prompt antimicrobial and anti-inflammatory functions, and induce immune cell recruitment, bacterial phagocytosis, and epithelial healing. The recently described lipopeptides, C14-R1 and C12-R2, have also shown AMP activity through the production of reactive oxygen species (ROS) (51). On the other hand, the barrier features of the mucus layer are completed with microbial metabolites produced by bacterial fermentation of dietary components, such as short-chain fatty acids (SCFAs). SCFAs regulate most intestinal epithelial cell functions, including cell turnover (52), mucus secretion by goblet cells (53), tight junction (TJ) proteins expression (54) and inflammasome- or hypoxia-inducible factor-mediated epithelial integrity (55, 56).

Below the mucus layer, the epithelial barrier maintains gut homeostasis and regulates immune responses (50). Epithelial cells are sealed by tight and adherens junctions (TJ and AJ, respectively), which are finely regulated by the influence of proinflammatory cytokines, and by signaling kinases and cytoskeleton, like myosin light chain kinases (5, 57). Intestinal epithelial cells possess a tightly regulated and specifically localized set of Pathogen-associated molecular pattern (PAMP) receptors and their signaling components for microbial detection. This innate immune-recognition equipment enables them to respond to microorganisms, thereby initiating the first steps in the host-pathogen interaction by regulating the immune response (58, 59). In addition, some studies unveil intestinal epithelial cells as non-professional antigen-presenting cells inducing inflammation or promoting tolerance (60–62). Following the detection of intestinal microbes through PAMP receptors, some specialized epithelial cells, such as goblet or Paneth cells (**Table 1**), respond generating molecules that protect mucosa from commensal microbes and invading pathogenic microorganisms, as mentioned above.

The Gut-associated lymphoid tissue (GALT) comprises both organized tissues such as lymphoid follicles, Peyer’s patches, and mesenteric lymph nodes (MLNs), where immune responses are induced, and scattered immune cells throughout the surface epithelium of the mucosa and in the underlying lamina propria, where effector functions are carried out (63).

In the steady state, neutrophils, located in the lamina propria, produce ROS and neutrophil extracellular traps (NETs) helping eliminating microbes translocated across the mucosal epithelium and facilitating tissue healing or immune cell recruitment (18). Eosinophils produce mediators including fibroblast growth factor (FGF)-2 and transforming growth factor (TGF)-β to promote tissue remodeling and repair (64, 65). In addition, mast cells, localizing along the gastrointestinal tract, differentially express PAMP receptors depending on their location and develop a fundamental regulatory and defensive function (26, 66). Intestinal macrophages remove senescent, apoptotic epithelial cells and promote epithelial integrity. They can also capture and destroy any bacteria that breach the barrier or send cellular processes across the epithelial barrier to sample luminal contents, which transfer to Dendritic cells (DCs) for presentation to T cells in the draining MLNs. Through their production of immunoregulatory cytokines, such as IL-10 and TGF-β, they maintain and facilitate secondary expansion of regulatory T cells (Tregs). In a similar manner, they support T helper (Th)17 cells and type 3 Innate lymphoid cells (ILC3) through their production of IL1-β (20). Also, differentially localized DC subsets can capture translocated IgA immune complexes (67) and continuously sample the lumen to establish a tolerogenic response to innocuous antigens (22). Finally, several innate lymphocytes like NK cells, invariant natural killer T cells (iNKT), and mucosal-associated invariant T (MAIT) cells have been identified as important in controlling gut immunity. Together with ILC3 and gamma delta (γδ)T, both iNKT and MAIT cells contribute to gut barrier integrity by producing IL-22, which is also important for promoting antibacterial defense (68–70) (**Figure 1** and **Table 1**).

Th cells control the entry of translocated antigens at the gut (71). However, the sustained activation and proliferation of the Th response is an impeller of chronic inflammation. On the other hand, regulatory T (Treg) cells in the gut secrete TGF-β that negatively regulates T cell function (41). Both Th17 and Treg subsets show reciprocal phenotype plasticity based on the environmental milieu (72). Any imbalance between these populations in a tissue highly exposed to bacteria such as the gut barrier leads to a dysregulated mucosal immune response, with consequences for liver regulation and disease (73, 74) (**Figure 1** and **Table 1**).

Just below the effector sites of the immune system layer, an additional cellular barrier, the GVB, controls entry into the portal circulation and access to the liver (75). Thus, if a molecule or a microorganism crosses the muco-epithelial barrier and escapes the local immune system, even to reach the systemic circulation the GVB must be disrupted. The GVB is characterized by the presence of TJ and AJ, which strictly control paracellular trafficking of solutes and fluids, together with other cell types, such as pericytes or fibroblasts, associated with the microvasculature and involved in the maintenance of the GVB, where they form a vascular unit.

### 1.2 The liver contributes to shaping gut immune landscape

Liver-derived molecules interact with the gut regional immune system defining tolerogenic or pathological interactions. In addition to metabolic functions, bile acids (BAs) are synthesized in the liver and incorporated by intestinal epithelial cells, exerting several immunomodulatory activities (76). Firstly, BAs induce anti-inflammatory responses through farnesoid X receptor (FXR) and Takeda G-protein-coupled receptor 5 (TGR5) on many tissues such as liver, intestine, or brain. Particularly, in macrophages, TGR5 activation inhibits nuclear translocation of nuclear factor κB (NF-kB), reducing the secretion of proinflammatory mediators such as TNF-α, IL-1β, IL-6, IFN-γ and nitric oxide (NO) (77, 78). Secondly, BAs interact with intestinal microbiota helping maintain its eubiotic state. In fact, a decrease in BAs levels may induce an overgrowth of pathogenic Gram-negative bacteria, with raised levels of lipopolysaccharide (LPS) (79) and subsequent induction of proinflammatory responses in the gut. Moreover, BAs such as deoxycholic acid have a direct impact on microbial membranes due to its hydrophobicity and detergent properties. Indirectly, BAs binding to FXR induces the control of gut microbiota through the production of intestinal antimicrobial molecules, such as angiogenin 1, that help control microbial overgrowth (80).

Together with BAs, IgA is the main isotype participating in microbial control in the gut-liver axis. Though main contribution on microbiota regulation is proportioned by sIgA produced by intestinal plasma cells, liver-derived IgA also contributes to the regulation of intestinal microbiota (81). Regardless the source, the sIgA patrols the intestinal mucus barrier, protecting epithelial cells and opsonizing luminal bacteria (15) without inducing a deleterious local inflammatory response (82). In fact, sIgA deficiency has been shown to disrupt the gut barrier *via* increased translocation of commensal bacteria (83).

The rest of metabolites and intermediaries drained by the liver into systemic circulation such as free fatty acids, choline and ethanol metabolites have also an impact on the physiological conditions of the gut, as they contribute to modulate the gut barrier interaction between the microbiome and the immune system (73).

### 1.3 The brain contribution in gut immune response

Gut-brain axis involves a bidirectional communication route with a cellular and molecular interchange modulated by threatening inflammatory insults. Intestinal microbiota and its products can directly modulate the central nervous system (CNS) immune profile (84). Though specific immune responses coming from CNS and controlling the gut remains still elusive, different mechanisms have been recently uncovered (85). On this point, systemic, neuronal, and cellular intercommunication pathways have been described. First, systemic proinflammatory elements can alter blood brain barrier (BBB), as it happens with GVB, leading to increased permeability and passage of inflammatory products along the axis. Thus, neuroinflammatory triggering by such products could give rise to potential intestinal tract immune responses, as glucocorticoids release under hypothalamic-pituitary-function adrenal (HPA) activation, can modulate intestinal functions (84) (**Figure 1**). Moreover, neuronal connection of the CNS with the gut allows the signaling communication and the inhibition of proinflammatory macrophages, as well as M cell function to control pathogen irruption and microbiota homeostasis (**Figure 1**). Additionally, dysbiosis induced by stress and intestinal Th17 production of IL-17A, has been described to feedback this stress response (86).

## 2 Altered gut-liver-brain axis in cirrhosis

### 2.1 Gut barrier dysfunction in cirrhosis

In cirrhosis, the intestinal barrier is markedly disturbed, prompting the passage of live bacteria and bacterial components or metabolites to the internal milieu in experimental models and patients (5, 16, 87). This derangement increases in severity as cirrhosis progresses (**Figure 1**).

Gut barrier dysfunction affects both physical (muco-epithelial and GV barriers) and immunological layers, and results in hyperpermeability of the whole intestine (5, 13, 88). Disrupted mucosal barrier integrity plays a pivotal pathophysiological role not only because it increases the risk of severe complications such as spontaneous infections, but also and more importantly, because it affects the natural history of liver disease and patient survival.

#### 2.1.1 Deranged muco-epithelial and gut vascular barrier permeability

Intestinal dysbiosis is the dominant player that sets the basis for epithelial barrier disruption. The gut microbiome in cirrhosis is characterized by reduced diversity and shows a significantly increased abundance of potentially pathogenic bacteria such as *Enterococcaceae*, *Staphylococcaceae* and *Enterobacteriaceae*, and a reduced relative abundance of potentially beneficial autochthonous bacteria such as *Lachnospiraceae* and *Ruminococcaceae* (89–91). Dysbiosis likely results from reduced bile flow with deficient levels of primary but increased levels of secondary BAs to the gut (92–94), which in turn results in reduced intestinal FXR signaling (10, 95), along with intestinal hypomotility. Intestinal dysbiosis and changes in BAs drive intestinal barrier functional abnormalities through changes in mucosal immunity and deficiencies in innate mechanisms of defense against bacteria (**Figure 1**).

The microbiome has been proposed to play a key role in mucus synthesis, release and barrier-function (96). In bile duct ligated (BDL) cirrhotic rats, there is lower mucus thickness, mucus weight per ileum length, and goblet cell numbers in the terminal ileum compared to healthy controls, predominantly with a reduction in mucin-filled goblet cells. This is associated with bacterial overgrowth in the inner most mucus layer (13). The closer proximity of bacteria to epithelial cells is one of the factors contributing to the passage of bacterial products in cirrhosis.

In humans and experimental models of cirrhosis, the observed structural changes in the small intestine include increased inter-enterocyte spacing with disorganization of TJ and AJ proteins, decreased intestinal mucosal proliferation and proliferation/apoptosis ratio, increased intestinal oxidative stress, edema of the lamina propria, infiltration by immune system cells, fibromuscular proliferation and a lowered villous/crypt ratio (5, 10, 21, 97–99). Diminished expression of zonula occludens-1 (ZO-1), occludin, claudin-1 and e-cadherin, and increased expression of claudin-2 have been reported in the small intestine of patients and experimental models of cirrhosis, especially in decompensated ones, with a concomitant increase produced in intestinal permeability, supporting the dynamic relationship between portal hypertension, gut bacterial translocation (GBT) and TJ and AJ expression in intestinal epithelial cells (5, 10, 21, 98). Portal hypertension results in intestinal mucosa hypoperfusion and hypoxia, which exacerbate oxidative damage in the gut mucosa (100). ROS can also enhance bacterial adhesion to epithelial cells and facilitate GBT across the mucosa (101). Increased cyclooxygenase-2 activity contributes to intestinal barrier disruption, as its inhibition increases ZO-1 and e-cadherin expression as well as intestinal permeability in rats with cirrhosis (102).

GVB is also profoundly altered in pre-clinical models of cirrhosis (13). This pathological endothelial permeability and accessibility in cirrhotic mice is associated with augmented expression of plasmalemma vesicle-associated protein (PV1), an integral membrane protein associated to the diaphragms of endothelial fenestrae, in intestinal vessels. Gut dysbiosis drives GVB disruption associated with liver disease, as mice receiving fecal microbiota transplantation (FMT) from high fat diet-fed mice, a pre-clinical model of non-alcoholic steatohepatitis, displayed increased PV1 expression in the small intestine compared to recipients receiving FMT from mice under control diet (103).

#### 2.1.2 Compromised antimicrobial host defense

Besides damage to the physical barriers, cirrhosis is also associated with compromised antimicrobial host defense, which concerns certain specialized populations of epithelial cells having particularly important roles in innate immune defense of the intestine, such as Paneth cells, as well as innate immune cells. In this sense, the loss of Paneth cell α-5, α-6 and α-7-defensins, lysozyme and Reg3γ AMPs has been described in experimental models and patients with cirrhosis (6, 10, 11, 30, 104). Interestingly, some of these changes have been associated with GBT in CCl_4_-induced ascitic cirrhotic rats. Moreover, a diminished activity against *E. coli* and *Enterococcus faecalis* in the distal ileum has been found in rats with cirrhosis with GBT compared with non-GBT (11). Of note, a negative correlation between α-5, and α-7-defensins expression and circulating endotoxin levels has been described in cirrhotic patients (104). Therefore, a deficiency in Paneth cell AMPs likely decreases mucosal killing activity with a consequent shift of the luminal bacterial composition associated with intestinal bacterial overgrowth and increased GBT in liver cirrhosis. In this regard, angiogenin-1 is an AMP transcriptionally regulated by FXR (105), which displays a selective bactericidal activity against Gram negative bacteria and modulates intestinal inflammation (106). Reduced ileum expression of angiogenin-1 has been observed in rats with CCl_4_-induced cirrhosis with ascites suffering GBT (10).

#### 2.1.3 Skewed gut innate and adaptive immune responses

Both structural changes and compromised antimicrobial host defense in the small intestine allow for bacterial colonization of the inner mucous layer and further promote the interaction of an altered gut microbiota, including an augmented bacterial burden and abundance of potentially pathogenetic taxa, with mucosal immune system cells. The consequence of this interaction between pathobionts and GALT is a state of subclinical inflammation with activation of innate immune cells, leading to proinflammatory cytokine and chemokine release, oxidative stress, and further recruitment of lymphocytes (11, 13, 21, 23, 107, 108).

Experimental studies have shown the increased expression of Toll-like receptor 4 (TLR4) and the proinflammatory cytokine TNF-α in the terminal ileum of CCl_4_-cirrhotic rats, especially in those with GBT (10, 109). In addition, the TNF-α levels in both the serum and multiple organs are significantly increased in CCl_4_-cirrhotic rats with compared with those without GBT (109) Similarly, patients with advanced cirrhosis with ascites exhibit elevated blood TNF-α levels along with an increase in the local production of TNF-α in the MLNs, which correlates with GBT (110). These findings suggest that, in cirrhosis, bacterial overgrowth and endotoxins significantly stimulate the secretion of TNF-α in the small intestine through the activation of TLR4. In the gut, immune dysregulation with a functional proinflammatory switch contributes to perpetuate intestinal barrier failure and GBT, both to the MLNs, and to the liver through the portal-venous route, due to disruption of the GVB (5, 13, 23, 107, 109). Subsequently, clearance deficiency by the cirrhotic liver allows bacteria and PAMPs getting into the systemic circulation (5, 13, 23, 107–109, 111). Recirculation of gut activated effector immune system cells along with inflammatory mediators, such as TNF-α, PAMPs and damage-associated molecular patterns (DAMPs) spreads the systemic inflammatory response inducing circulatory and remote organ dysfunction (91, 107, 109–113) (**Figure 1**).

Regarding to innate immune cells, a deranged innate immune response has an important contribution to the gut barrier disruption that occurs in cirrhosis patients and experimental models and includes: i) activated CD14+Trem1+inducible nitric oxide synthase (iNOS)+ macrophages, secreting IL-8, CCL2, IL-6 and NO, which correlate with an increased expression of claudin-2 (21); ii) an increased number of activated CD103+ DCs producing TNF-α and showing higher phagocytic and migratory abilities, when GBT is not present, which switch to a non-activated phenotype, and relatively deficient function when cirrhosis progresses and GBT occurs, likely facilitating bacterial passage (23); iii) the expansion of IFN-γ-secreting γδT- and NK cells, that could be related with TJ disorganization (5); and iv) a reduced IL-22 production by ILC3s which promotes diminished intestinal Reg3γ expression, resulting in GBT to the liver (30) (**Figure 1** and **Table 1**).

Changes in the small intestine’s adaptive immune response are also prominent especially as cirrhosis progresses to the ascites stage, and consist of: i) high infiltration of several activated Th and cytotoxic T (Tc) CD8αβ+ cell subsets (5, 109); ii) high proportion of Th1 and IFN-γ-producing Tc cells, which directly correlates with gut permeability that is probably due to endocytosis of TJ proteins (5, 114); iii) Th17 depletion, which also contributes to barrier damage as IL-17 play a role in intestinal epithelium homeostasis by inducing TJ function and AMPs production (115); iv) increased numbers of Tregs in a likely response to bacterial challenge, although they seem unable to offset the proinflammatory immune response; and v) an increased number of B cells, which contrasts with the reduced synthesis of IgA in the jejunum of rats with cirrhosis and the diminished fecal IgA content of the intestine in cirrhotic rats (5, 16, 17) (**Figure 1** and **Table 1**).

Intestinal dysbiosis leads to a dysregulation of the innate and adaptive immune responses of the intestinal mucosa that further contribute to bacterial mucosal adhesion and colonization, as well as intestinal hyperpermeability. As cirrhosis progresses from the preascitic to the ascitic stage a dysregulated mucosal immune system contributes to the derangement of epithelial TJ, reduced secretion of AMPs and impaired phagocytic function of DCs, which facilitate the increased passage of bacteria and bacterial products to the systemic circulation (**Figure 1** and **Table 1**).

The key pathogenetic role of bacteria is confirmed by the reversibility of most intestinal immune abnormalities and increased gut permeability provoked by antibiotic-induced microbiome reorganization (5, 23). Consistently, antibiotics reduce immune cell infiltration in the intestinal mucosa of cirrhotic animals. Of note, bowel decontamination normalizes the frequencies of proinflammatory IFN-γ and TNF-α cytokines, the phagocytic activity of DCs, and gut permeability, as revealed by a reduction in fecal albumin loss and by fully suppressed GBT in cirrhotic rats with ascites (5, 23).

### 2.2 Disturbed gut-brain function during cirrhosis

A mild neurocognitive dysfunction is present in as much as 80% of patients with cirrhosis. This condition, named minimal or covert hepatic encephalopathy (HE) affects quality of life (116) and predicts the development of overt HE (117). This complication encompasses a wide range of cognitive, psychomotor and psychiatric disturbances that heralds a poor prognosis with negative impact on health-related quality of life, liver transplant priority and patient survival (118, 119).

From a neurologic point of view, hepatic encephalopathy in cirrhosis is primarily astroglial in nature, characterized by Alzheimer type 2 astrocytosis together with activation of microglia indicative of neuroinflammation (120, 121). Inflammation and hyperammonemia are associated with enhanced cognitive impairment in patients with cirrhosis (122). It has been proposed that inflammation synergistically acts with ammonia in driving nitrosation of brain proteins in BDL cirrhotic rats (123). Proinflammatory cytokine levels are also increased in patients with covert hepatic encephalopathy (124), and these inflammatory mediators have been correlated with serum ammonia in patients with extrahepatic portal venous obstruction (125). The relevance of the neutrophil response in the pathogenesis of hepatic encephalopathy has also been documented (126), and proinflammatory IL-1β and IL-6 have been correlated with neurocognitive scores and with health-related quality of live questionnaires in patients with cirrhosis (127) (**Figure 1**).

A link between altered flora (higher *Veillonellaceae*), poor cognition, endotoxemia, and inflammation (IL-6, TNF-α, IL-2, and IL-13) has been described in cirrhotic patients with compared with those without HE. In addition, in the cirrhosis group, *Alcaligeneceae* and *Porphyromonadaceae* positively correlated with cognitive impairment (128). An increased risk of cognitive impairment in patients with cirrhosis and bacterial infections has been reported, including those with subclinical cognitive alterations (129). In this latter group of patients, even bacterial antigen translocation, without the need of an over infection, is associated with increased serum ammonia and NOx levels, and a more severe neurocognitive condition (130).

Finally, the relationship between Th17 cells and cognitive impairment has also been documented. Th17 cells have been described to cross the BBB and promote brain injury in neurological diseases (131). Systemic inflammation associated with cirrhosis may therefore increase BBB permeability and facilitate brain T cell recruitment in the context of advanced liver disease (**Figure 1**).

## 3 Therapeutic approaches targeting the intestinal homeostasis in cirrhosis

Abnormalities in microbiota composition and bacterial overgrowth set the stage for the gut-liver axis disruption with intestinal barrier failure, which is distinctive of cirrhosis. These facts enable increased passage to the bloodstream of bacteria or their products, which promote systemic inflammation and bacterial infections, and worsen cirrhosis progression. The disrupted gut-liver axis observed in cirrhosis is a topic of active research, considering its contribution to disease progression. We thereby briefly summarize novel therapeutic approaches to target the gut-liver axis aimed to halt cirrhosis progression.

### 3.1 Antibiotics

The effects of bowel decontamination with poorly absorbable antibiotics are the redistribution of microbiota composition, reduced intestinal permeability and GBT, and improved proinflammatory activation of mucosal and circulating immune cells along with circulatory dysfunction in human and experimental models (5, 112). Norfloxacin and ciprofloxacin are standard of care for primary and secondary prevention of spontaneous bacterial peritonitis, and norfloxacin reduces the risk of infection and 6-month mortality in patients with advanced cirrhosis (132, 133). Rifaximin is a broad-spectrum compound that improves and prevents overt hepatic encephalopathy and ameliorates systemic inflammation and endotoxinemia in patients with cirrhosis and encephalopathy (134, 135). Rather than promoting bowel decontamination, rifaximin exerts its effects by eliminating polarization of the gut microbiome *via* suppression of mucin-degrading species rich in sialidase and known to induce gut barrier damage while preserving beta-diversity, e.g. *Streptococcus* and *Veillonella spp* (135). Rifaximin associated with simvastatin to prevent Acute-on-Chronic Liver Failure and reduce complications in decompensated cirrhosis is being currently tested in clinical trials (NCT03780673). Alternatives to antibiotics are a topic of research as their long-term use has been linked to reduced bacterial diversity and multidrug-resistant microorganisms (136).

### 3.2 Probiotics and diet

Probiotics displace resident dysbiotic bacteria and reconstitute a healthy microbiome. *Bifidobacterium pseudocatenulatum* CECT7765 induces a shift towards an TLR2-mediated anti-inflammatory cytokine profile in intestinal lymphocytes and macrophages, improves gut barrier integrity, reduces intestinal permeability, and decreases bacterial DNA translocation, serum endotoxin levels and burden of bacterial antigens in the liver in cirrhotic patients and experimental models (137–139). *Lactobacillus rhamnosus GG* supernatant therapy also increases mRNA expression of TJ proteins and reduces intestinal permeability in a mouse model of chronic-binge alcohol feeding. This therapy led to a decrease of GBT to the liver and an overall balance restored of Tregs, Th17s, and IL-17 (140, 141). Furthermore, probiotic supplementation has shown significantly increase serum neopterin levels and the production of ROS by neutrophils (142). These findings might explain the beneficial effects of probiotics on immune function, as well as in ameliorating gut barrier dysfunction in cirrhosis. However, studies on the impact of probiotics on cirrhosis complications and progression, including hepatic encephalopathy, have severe methodological limitations and patient numbers have been too small to draw valid conclusions. In parallel, an international study has shown that a diet rich in coffee, tea, fresh vegetables and fermented milk is associated with an increased diversity of microbiota species and reduced number of hospitalizations in patients with cirrhosis, suggesting the benefits of dietary modulation of the gut microbiome in favor of liver health (143).

### 3.3 Gut molecular adsorbents

Another therapeutic approach is to reduce the amount of bacterial toxin being absorbed from the intestine in patients and experimental models with chronic liver disease. In contrast to the strategies based on the prolonged use of antibiotics that reduce intestinal bacterial populations, use of scavengers of bacterial toxins has the advantage of avoiding the risk of establishing antimicrobial drug resistant intestinal bacteria. Yaq-001 is a non-absorbable activated charcoal with a high adsorptive capacity for endotoxins and other products of bacterial metabolism preventing their absorption into the bloodstream. Evidence in BDL rats showed that Yaq-001 was associated with shifts in microbiome composition, attenuated the LPS-induced production of ROS by monocytes, and reductions in liver injury and portal pressure (144). A phase-II clinical study in patients with cirrhosis and diuretic-sensitive ascites treated with Yaq-001 or placebo for 12 weeks suggested proof of a mechanism that Yak-001 modulated systemic endotoxinemia and inflammation by improving gut inflammation and its permeability (NCT03202498).

### 3.4 FMT

FMT restores a healthy gut microbial environment and physiological colonization *via* different routes of administration. Microbiota transplantation in patients with recurrent hepatic encephalopathy proved safe in the long-term and has shown to rescue antibiotic-associated disruption in microbial diversity, improve cognitive function, and reduce encephalopathy recurrence and hospitalizations (145). These studies have involved a limited number of patients and efficacy trials are pending. In this regard, FMT is currently being tested in a randomized controlled trial in patients with decompensated cirrhosis to address survival and liver related outcomes (NCT04932577).

### 3.5 FXR agonists

Diminished luminal BA availability in cirrhosis provokes a reduction in intestinal FXR signaling which seems, at least partly, to mediate the gut barrier disruption in cirrhosis, as FXR-agonists reduce GBT *via* the portal-venous route to the liver (13). In experimental cirrhosis, FXR activation by obeticholic acid (OCA) increases ileal protein expression of the main TJ proteins and AMP secretion (10, 13), influences intestinal epithelial cell proliferation and apoptosis (146), and exerts potent antiinflammatory actions in the intestine, stabilizing epithelial integrity (10). Herein, OCA impacts the mucous machinery by increasing ileal goblet cell numbers in cirrhotic animals (13). On the vascular side, the FXR agonist OCA stabilizes the dysfunctional GVB in experimental cirrhosis (13).

### 3.6 Administration of IL-22

IL-22, a cytokine member in IL-10 family, is mainly secreted by various immune cells, such as ILCs and MAIT cells, which targets epithelial cells in several organs, including the intestine and the liver. IL-22 promotes survival and proliferation of hepatocytes and liver progenitor cells, thereby promoting liver repair (147). Additionally, IL-22 increases the expression of intestinal Reg3 lectins, which maintain low bacterial colonization of the inner mucus layer and reduce GBT to the liver. In a mouse model of ethanol-induced liver disease, intestinal IL-22 has the beneficial effect of reducing GBT in the intestine by increasing the expression of Reg3γ. Bacteria engineered to produce IL-22 in the intestine (without increasing systemic IL-22) ameliorate experimental ethanol-induced steatohepatitis *via* induction of Reg3γ (30). Importantly, due to this combination of protective effects in the liver and in the intestinal barrier IL-22 is a promising drug for the treatment of alcoholic hepatitis.

## 4 Concluding remarks

While the gut immunity during homeostasis plays a crucial role in liver and brain functions, mainly through its interaction with symbiotic microbiota, the progression of advanced chronic liver disease significantly compromises the immunological behavior at the gut and brain barriers of cirrhotic patients. Therefore, therapeutic options aimed at restoring gut immune homeostasis in these patients are of relevance to prevent cirrhosis-derived complications. However, despite intense experimental research, its translation into clinic remains a lengthy goal.

## Author contributions

LM, EC, AA, RF: manuscript design, writing and final version approval.
